# A Pre–Post Study on the Cardiorespiratory Response to Different Protocols of Exposure on a Vibratory Platform in Young Healthy Individuals

**DOI:** 10.3390/ijerph19084668

**Published:** 2022-04-12

**Authors:** Elena Ioana Iconaru, Manuela Mihaela Ciucurel, Luminita Georgescu, Mariana Tudor, Monica Marilena Tantu, Constantin Ciucurel

**Affiliations:** 1 Department of Medical Assistance and Physical Therapy, University of Pitesti, 110040 Pitesti, Romania; mariana.tudor@upit.ro (M.T.); monica.tantu@upit.ro (M.M.T.); constantin.ciucurel@upit.ro (C.C.); 2 Department of Psychology, Communication Sciences and Social Assistance, University of Pitesti, 110040 Pitesti, Romania; manuela.ciucurel@upit.ro; 3 Department of Physical Education and Sport, University of Pitesti, 110040 Pitesti, Romania; luminita.georgescu@upit.ro

**Keywords:** whole-body vibrations, protocols of stimulation, acute effects, cardiorespiratory parameters

## Abstract

This study aimed to investigate the acute specific physiological effects of 15 min of whole-body vibration (WBV) exposure at six different types of vibrations on cardiorespiratory function in 26 healthy young subjects (sex ratio, 1:1; mean age, 20.73 years). The protocols included six variants of a combination of mechanical stimuli with different frequencies (15, 25, and 35 Hz) and direction of stimuli (vertical or diagonal). The investigated cardiorespiratory parameters were heart rate (HR), arterial oxygen saturation (SaO_2_), respiratory rate (RR), and spirometric indicators: tidal volume (TV), vital capacity (VC), forced vital capacity (FVC), forced expiratory volume at 1 s (FEV_1_), and maximum voluntary ventilation for 12 s (MVV). The data series were statistically processed by using descriptive and inferential statistical methods: the Shapiro–Wilk test, the two-way ANOVA with repeated measures, and post hoc analysis. We obtained significantly higher values for HR, TV, VC, FVC, FEV_1_, and MVV after the WBV exposure. These parameters are significantly influenced by both the frequency and direction of stimuli, and certain protocols of WBV are noticeable for their distinct effects. Our results offer a new perspective on the possibility of using preferential variants of vibratory stimulation to obtain maximum cardiorespiratory physiological effects.

## 1. Introduction

### 1.1. The Principle of Whole-Body Vibration and Its Application in Medicine

Whole-body vibration (WBV) uses low-magnitude, high-frequency mechanical stimuli, as generated by a vibrating platform, that are transmitted through the human body and stimulate all cells [1]. The oscillatory motion is characterized by amplitude (peak-to-peak displacement, in mm), frequency (repetition rate of vibrations waves, in Hz), and the magnitude of the effect generated, the peak acceleration (a peak, in g, defined as maximal vertical acceleration within a cycle) [2,3]. The WBV programs can be applied for different exposure durations and directions of exposure (vertical, horizontal or diagonal vibrations), presently without any clear standardization of the recommended protocols in various clinical settings [4,5]. Applying vibrations to the human body for therapeutic purposes falls within the methods of mechano-therapy, rhythmic neuromuscular stimulation, and biomechanical stimulation or cyclic massage. WBV platforms are designed to deliver vibrations programs across a range of frequencies (15–60 Hz) and displacements from 1 to 15 mm [2]. Recent studies have demonstrated that the frequency of vibratory stimuli with trophic potential effects on tissues is in the range of 20–50 Hz [6].

The use of vibration platforms is historically related to research that has proven its utility in the prevention of sarcopenia and osteoporosis in the case of human exposure to microgravity [7], immobilization syndrome, or in the context of menopause/aging [5]. Moreover, many authors have described the positive effects of vibrations in improving musculoskeletal function [8,9], increasing sports performance in terms of flexibility and muscle strength [7,10], and optimizing of postural stability [11]. At present, there are numerous and various devices that use this principle of sinusoidal mechanical oscillations, with wide applicability in the field of medical rehabilitation, aerospace medicine, sports performance, and wellness.

In regard to the dynamics of the WBV effects on the human body, the scientific literature reveals immediate and short-term effects, as well as respective long-term training effects. According to certain opinions, the reported functional acute effects are less significant than the chronic ones [12]. Moreover, the results obtained by repeating the procedures are dependent on the duration of the training program and the individual characteristics of the subjects [11]. The reported effects are multisystemic, being the subject of multiple researches. Thus, cellular and molecular effects have been put into evidence in regard to musculoskeletal trophicity’ improvement of neuro-hormonal status; and metabolic effects, such as anabolic stimulation, cardiovascular, respiratory and nervous system effects, antiaging effects, antialgic effects, etc. [2,13,14].

### 1.2. The Effects of Whole-Body Vibration on the Cardiorespiratory System and New Perspectives for Investigation

We have identified relatively few studies from the specialized literature on the acute effects of vibrations on the cardiorespiratory system in healthy individuals. Regarding the heart, studies focused especially on the effects of WBV on heart rate variability, such as acute responses and training adaptations [15,16]. Moreover, some research investigated the cardiovascular physiological effects of WBV on heart rate and blood pressure [4,17], or of the combination of WBV and exercise exposure on hemodynamic parameters [18]. Other relevant papers investigated the acute respiratory effects of the manual vibrations exerted on the thoracic wall, with or without compression, specific to the animal or human physiotherapy procedures [19,20]. Furthermore, recent studies focused on the chronic effects of the physical exercise programs associated with WBV protocols during pulmonary rehabilitation.

For the respiratory studies, it should be noted that most clinical trials included patients with chronic obstructive pulmonary disease (COPD), cystic fibrosis, or pulmonary arterial hypertension; patients after lung transplantation; or mechanically ventilated patients in the intensive care units. These studies aimed on the therapeutic effectiveness of the interventions, in addition to targeted medical treatment, in terms of symptomatic and functional improvement, increase of patients’ effort capacity and quality of life, reduction of drug doses, etc. [13,21,22,23,24,25]. In the abovementioned studies, gasometric parameters, and cardiopulmonary adaptation to the effort (oximetric indexes, maximum ventilation, level of dyspnea during exercise, effort capacity, etc.) were, in particular, assessed. It is important to add that no adverse effects of the procedure were reported in such studies [26], even though a known risk exists for harmful effects on the human body when inappropriately used [12]. Nevertheless, the quoted studies have focused on the effects of the association of the static or dynamic exercise with WBV, and in these cases, supplementing exercises with vibration leads to an increase in oxygen uptake [27]. In addition, the results can be significantly affected, since categorical variables, such as the specific pathophysiological context of respiratory morbidity, certain age groups (usually adult or elderly patients), or/and associated pharmacological treatments, are considered. That is why it is difficult to quantify only the vibratory mechanical component in outcomes determinism. Last but not least, some authors deny the existence of the chronic beneficial effects of exposure to WBV in the pulmonary function [26].

We can affirm that there is a niche of investigation of the spirometric indicators (such as tidal volume—TV, vital capacity—VC, forced vital capacity—FVC, forced expiratory volume at 1 s—FEV_1_, and maximum voluntary ventilation—MVV) in the physiological context of the acute adaptation of the human body to WBV exposure. As a result, it becomes a challenge to understand these adaptations and to refine the interpretations of the effects of various WBV protocols in regard to duration, frequency, and spatial orientation of mechanical stimuli. Thus, if we put into evidence these acute cardiorespiratory effects, based on clinical evidence, we can then better understand the chronic effects, which justify the WBV utility in respiratory pathology. In the absence of standardization of certain intervention protocols, which is the major issue of this rehabilitation technique, our results could ensure a further restructure of the WBV programs in clinical practice.

## 2. Materials and Methods

### 2.1. Aim of the Study and Premise

This study aimed to investigate the acute specific physiological effects of WBV exposure on cardiorespiratory function in the healthy human body. Therefore, we were interested in determining the cardiorespiratory effects of different type of WBV programs (such as frequency and direction of exposure to mechanical stimuli), but with the same temporal pattern (a session duration of 15 min), applied to young subjects. The problem is of great relevance, since there is still insufficient information on the mechanism by which these procedures interfere with the cardiorespiratory homeostasis, and they may become beneficial in medical practice as associated therapies.

### 2.2. Participants and Type of Study

An interventional before-and-after study was performed on a cohort of 26 healthy young people (sex ratio, 1:1; mean age, 20.73 years). We investigated the acute cardiorespiratory effects of 15 min of WBV exposure at six different types of vibrations (in terms of combination of frequency and directions of application of stimuli) in the group of subjects, with each subject being his or her own comparator. Participants were students at the University of Pitesti who voluntarily accepted to be included in the research. The study was approved by our institutional ethical committee (registration number 950/04.05.2018), and all participants provided informed consent by signing an agreement form to participate in this research. The inclusion criteria were age between 19 and 22 years, good health (no significant pathological history, without smoking habits, normotensive, non-obese, without any medication in last two weeks), confirmed by a medical examination, no history of training on vibration platforms or exposure to vibrations in occupational or leisure activities, and the absence of contraindications for testing. Exclusion criteria were recent antecedents of musculoskeletal, respiratory, cardiovascular, or neurological disorders and any form of acute or chronic pain.

### 2.3. Data Acquisition

The experiment took place in the laboratory facilities at the University of Pitesti. Firstly, we assessed anthropometric parameters for all participants (body height—H, in cm; weight—W, in kg; and body mass index—BMI, in kg/m^2^), according to standard procedures. Next, 6 successive sessions of testing (heart rate, pulse oximetry, respiratory rate, and spirometry) were performed for the experimental group. Therefore, we realized for each subject during each session of testing an initial assessment, before the vibration procedure, and a final one, after a different protocol of 15 min of exposure to WBV. The WBV protocols included 6 variants of combination of mechanical stimuli: with different frequency—low-frequency vibration (15 Hz—f1), medium-frequency vibration (25 Hz—f2), and high-frequency vibration (35 Hz—f3); and direction of stimuli—vertical (v) or diagonal (d). For this purpose, we repeated the evaluation for each subject in six non-consecutive days.

The assessments were carried out between 8 a.m. and 11 a.m. for all subjects, under conditions of body thermal neutrality (environmental temperature inside the laboratory of 22–26 Celsius degrees, humidity under 80%, subjects wearing lightly sports outfit). All measurements were performed under strict standard conditions, at rest, at least 30 min after food intake. Arterial oxygen saturation (SaO_2_, %) and heart rate (HR, beats/min) were measured by a portable fingertip pulse oximeter and heart-rate monitor (CMS 50-DL ContecTM, Qinhuangdao, China), a device with good references for research studies [28]. The recording of SaO_2_ and HR was performed in roughly ten seconds for each participant. Respiratory rate (RR) was recorded for one minute, as the number of breathing cycles (breaths/min), by direct observation of the chest movements. To obtain real values of the RR, subjects were not informed about the aim of this respiratory assessment. For SaO_2_, HR, and RR, subjects sat in a comfortable position on a chair, with the spine extended, resting-state, eyes open, and no speaking conditions [29].

For spirometry, subjects adopted a sitting-straight position on the chair, with the neck in a neutral position, eyes open, and a nose clip applied at the nostrils. The operator was in an adequate position to assist the subject and to see the display of the digital device. Spirometry was performed with a Spiro Analyzer ST-75 Fukuda Sangyo Co., Ltd., Tokyo, Japan, with PC-based software (Data Management Software FS/PC kit), according to the European Respiratory Society and American Thoracic Society guidelines [30]. The device was calibrated before the session of measurements, and each subject performed two consecutive rounds of testing (separated by a pause of 1 min) for VC, FVC, and MVV. From the acceptable series of data, we took into consideration the best result for each participant. After exporting the records, we considered for data analysis the following parameters: TV, VC, FVC, FEV_1_, and MVV. The duration of measurements was 50 s for VC, 25 s for FVC, and 12 s respectively for MVV.

Subjects from the experimental group were exposed to WBV protocols by using a SALUSSSTAR^®^ Innoplate platform, designed by Saluto, a manufacturer of Maxline Produkt, Design GmbH, Austria (medical device according to 93/42/EEC directive). The platform can generate multidimensional, vertical, or diagonal vibrations, and the frequency of the mechanical stimuli range from 15 to 35 Hz is adjustable in one step [31]. The vibration amplitude varies from 0.5 mm in the center of the platform to 4 mm in its outer parts. The programmable duration can be set between 1 and 20 min. As a working methodology, each participant was instructed to adopt an orthostatic position, with the plants slightly diverging in the center of the platform, with bilateral palmar support on the anterior support bar, and with elbows in flexion at 90 degrees.

The most important issue in the case of interventional studies in terms of reliability refers to the process of randomization of subjects. Many studies focusing on the acute effects of WBV on functional parameters, with one-group pretest–posttest design, are based on randomization of intervention protocols [15,17,18]. Thus, in our case, subjects were exposed in successive nonconsecutive days to six sessions of multidimensional WBV, according to the following protocols in relation to the frequency and direction of application of the stimuli: f1v, f2v, f3v, f1d, f2d, and f3d. On the day of rest, the subjects were advised to have a normal diet and physical activity regime, without any form of excess. The order of sessions of testing was randomized by drawing lots, and the interval between trials was sufficient to allow for residual effects to dissipate. The selected duration for each protocol of continuous stimulation was 15 min. This value is consistent with the views of specialists on the use of vibrations for maximum effects, in the absence of possible side effects [4,32]. Therefore, the acute differences in cardiorespiratory parameters were investigated by using a randomized crossover design with six conditions. Another important element that was taken into account to ensure reliably for unidentifiable differences between subjects was the standardization of test conditions. Thus, the same standardized protocols were assured to minimize differences in participants’ daily routines and provide unbiased estimates of the effects of specific interventions.

We mention here the fact that, in clinical practice, most of the WBV programs are usually implemented in sessions of 4–20 min and can be repeated every day for months in order to obtain long-lasting effects [33,34]. Obviously, the frequency, duration, and direction of stimuli application determine the training load of WBV and, thereby, the possible physiological adaptations. We were especially interested in highlighting acute systemic cardiorespiratory effects; therefore, we opted for the above methodology.

### 2.4. Outcomes and Statistical Analysis

Following the evaluations of the group of subjects, we recorded a series of data, which were statistically processed in a first step by using descriptive statistics (univariate analysis for determining of mean, standard deviation (SD), and coefficient of variation (CV). Next, we considered it necessary to put into evidence if the series of data follows a normal distribution pattern, and we applied the Shapiro–Wilk test. For the series of data with non-normal distribution, we applied a natural logarithmic (log) transformation to best meet the assumption of normality [35]. In order to compare the difference between means, we applied, as a parametric inferential statistical method, the two-way ANOVA, with repeated measures, using SPSS statistics 20.0. Therefore, the dependent variables were, at a time, the cardiorespiratory parameters (HR, SaO_2_, RR, TV, VC, FVC, FEV_1_, and MVV), whilst the two factors were the “conditions” (exposure to 6 different types of WBV, as frequency and direction of stimuli) and “time” (before and after exposure). The Mauchly’s test for the sphericity was used in order to control for within-group variance and, when necessary, the Greenhouse–Geisser correction to combat the violation of the assumption of sphericity. Next, we applied the Tests of Within-Subjects Effects to determine the statistically significant interaction term. For the Tests of Within-Subjects Effects, we also calculated the Partial Eta Squares for effect size and the Observed Powers to detect the power of the study for the given sample size. Finally, post hoc multiple pairwise comparisons (Bonferroni Test) were performed when the ANOVA results were significant. The level of statistical significance was set at *p* < 0.05. In the interpretation of the results, if the *p*-value was larger than 0.05, then we had confirmed the null hypothesis, H_0_, which assumes there is no difference between the groups regarding the investigated parameters.

## 3. Results

**Figure 1:**
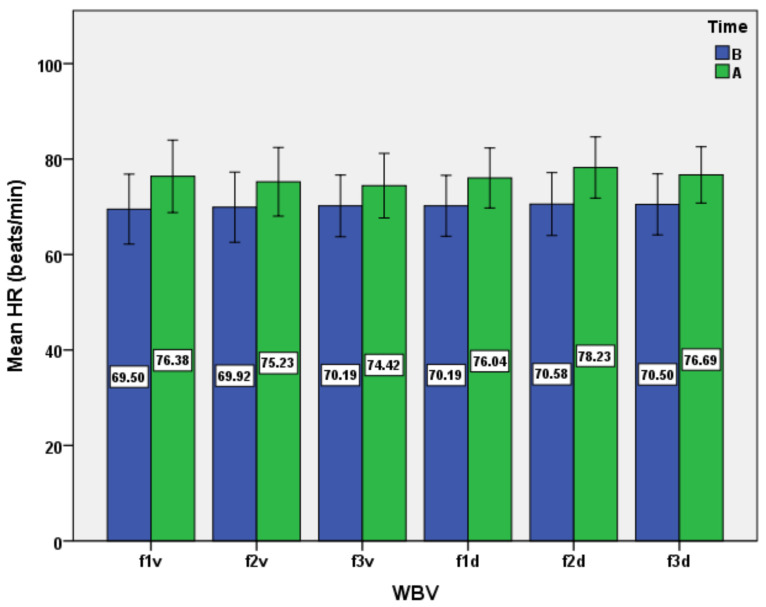
Impact of the WBV exposure on heart rate (HR) for the six protocols (means and standard deviations). B, before; A, after.

**Figure 2:**
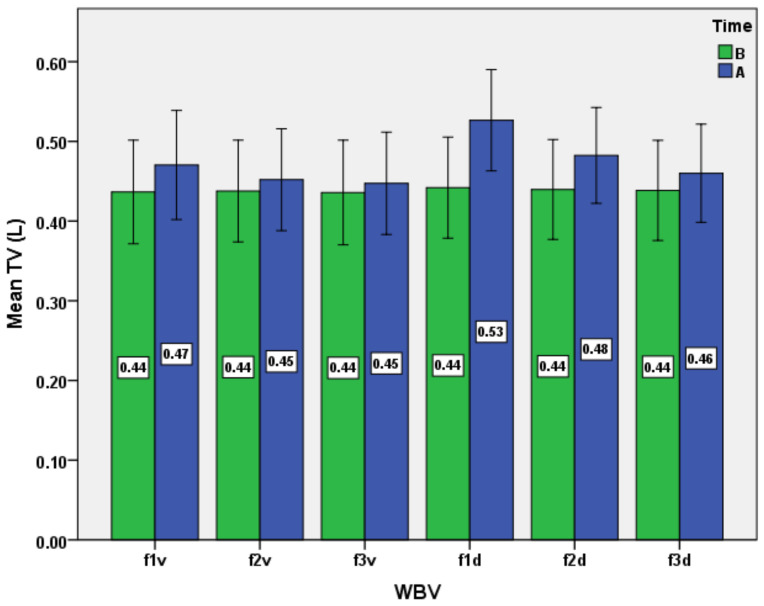
Impact of the WBV exposure on tidal volume (TV) for the six protocols (means and standard deviations). B, before; A, after.

**Figure 3:**
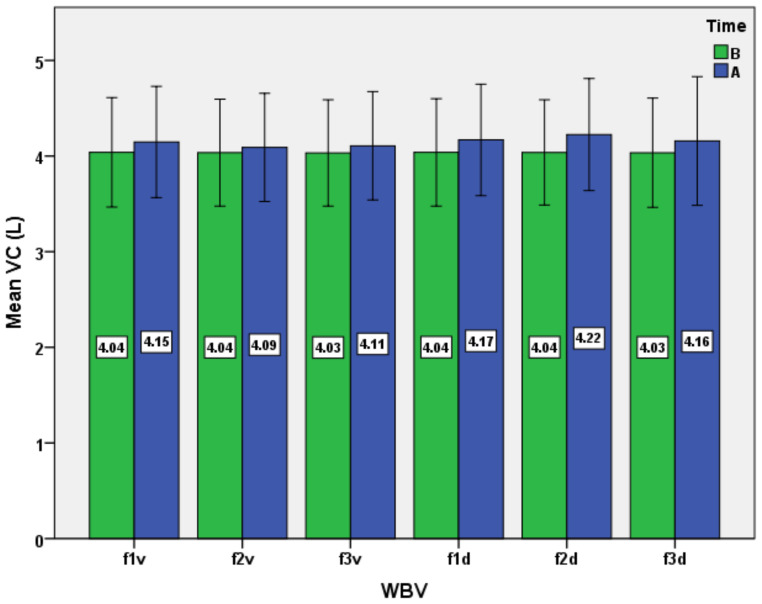
Impact of the WBV exposure on vital capacity (VC) for the six protocols (means and standard deviations). B, before; A, after.

**Figure 4:**
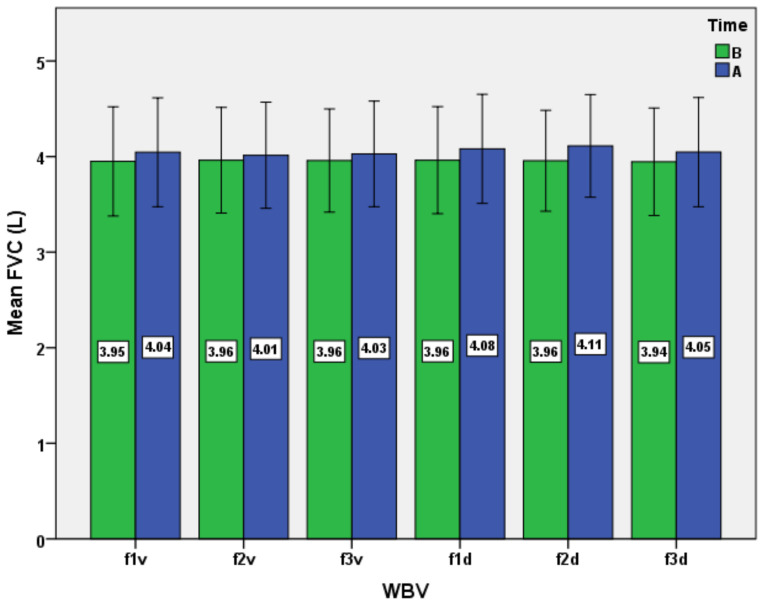
Impact of the WBV exposure on forced vital capacity (FVC) for the six protocols (means and standard deviations). B, before; A, after.

**Figure 5:**
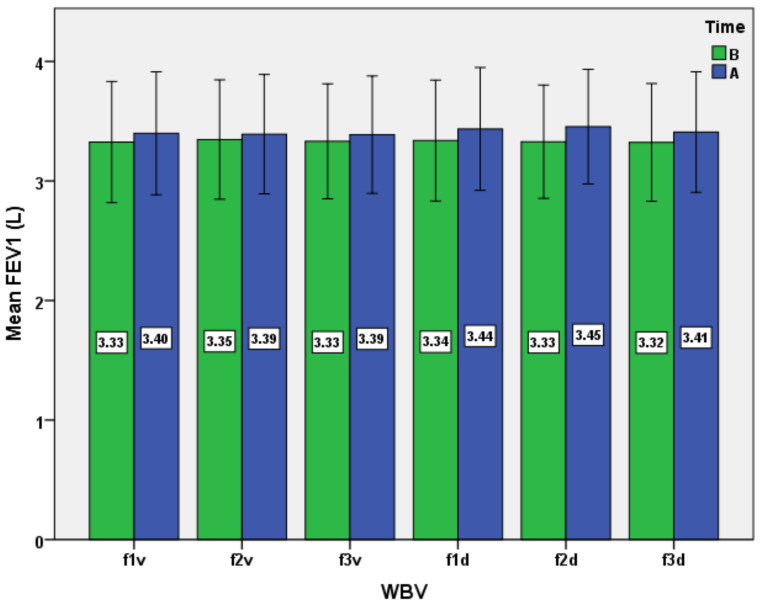
Impact of the WBV exposure on forced expiratory volume at 1 s (FEV_1_) for the six protocols (means and standard deviations). B, before; A, after.

**Figure 6:**
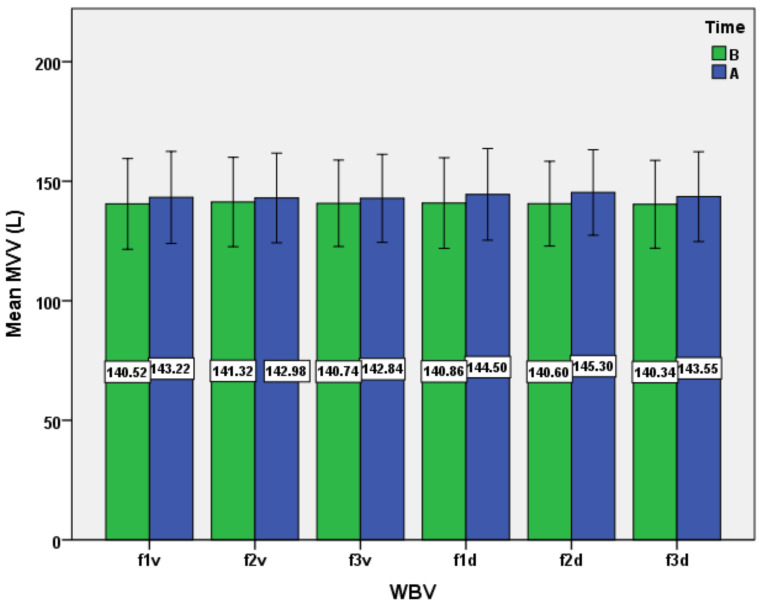
Impact of the WBV exposure on maximum voluntary ventilation (MVV) for the six protocols (means and standard deviations). B, before; A, after.

The results consist of several datasets with quantitative variables, which correspond to the distinct evaluations of the experimental group. The results are presented as mean ± standard deviation in Table 1 and Table 2 and Figure 1, Figure 2, Figure 3, Figure 4, Figure 5 and Figure 6.

We mention that the variables age, weight, and height had a non-normal distribution after applying the Shapiro–Wilk test. Moreover, after applying the same test, the normal data distribution only for HR and TV was confirmed. For the other variables, a natural logarithmic transformation was completed in order to reach the normality required. For SaO_2_ and RR, as exceptions, the log transformation was not effective for establishing normality. Therefore, the inferential statistical analysis for these variables was inapplicable. As a general remark, we found all respiratory baseline parameters for all subjects to be in normal ranges (over 100% of the predicted values, depending on age and gender for TV, VC, FVC, FEV_1_, and MVV). The ANOVA analysis for all subjects, for the respiratory variables, including the type of WBV exposure (six different variants as frequency and direction of stimuli) and time (before versus after) showed the main effect of time, WBV exposure, and the time*WBV exposure interaction (Table 3 and Figure 1, Figure 2, Figure 3, Figure 4, Figure 5 and Figure 6).

We can observe that most of the main effects (for time and WBV) and interactions (time*WBV) are significant, at *p* < 0.05, with Partial Eta Squares indicating large effect sizes (values higher than 0.13) [36]. In addition, the Observed Powers were all greater than 80% for most of the statistically significant situations, and, therefore, we can conclude that the sample size was adequate. The Bonferroni post hoc analysis in pairwise comparisons (Table 4) suggested statistically significant differences between the conditions “before and after” WBV exposure and some of the different types of stimuli. This indicates that certain combinations of frequency and direction of stimuli produce distinct effects on cardiorespiratory parameters.

## 4. Discussion

There are numerous studies related to the acute and chronic effects of vibrations on the human body that analyze different types of WBV protocols utilized, such as the frequency and direction of application of the stimuli. In fact, most research in the field mainly targets the effects of the vibrations on tissues, using vertical or horizontal stimuli. In the present study, as a distinctive element, we compared the acute cardiorespiratory effects obtained by application of different frequencies of vertical and diagonal vibratory stimulation. Thus, from the inferential perspective, we can draw a number of applicative conclusions by referencing the recorded parameters.

To ensure the internal validity of the results, we considered the study design, as a whole, a way of manipulating the independent variable (WBV protocols), and the random applications of vibratory stimulation protocols in the group of subjects. As for the external validity of results, we took into consideration the total involvement of subjects and evaluators in conducting the experiment, building the study group by rigorously applying the criteria for inclusion and exclusion of subjects, and avoiding situational disruptors during testing. We also selected reliable methods of measuring and manipulating variables, and we applied some consecrated inferential statistical instruments to ensure the statistical conclusion validity. All of these elements allowed us to highlight reliable evidence concerning the cause-and-effect relationships for our data.

Firstly, by analyzing the HR dynamics during the series of tests (Figure 1), we noticed that, for all protocols of WBV application, there was a slight mean increase in HR after exposure to WBV, with statistical significance (Table 3). The inferential perspective also suggested statistically significant differences in the post hoc analysis only between f3v and f2d protocols (Table 4). Although we cannot extrapolate the other results, we can only observe that the highest mean increase of HR occurs after the diagonal stimulation, at the frequency of 25 Hz. On the second place is the mean increase of HR at the vertical stimulation at 15 Hz. The results can be explained by the fact that, in the case of vertical stimuli, the resonant frequency of the viscera is generally below 20 Hz [34], and of the heart at 4–8 Hz [37]. In the case of the orthostatic position, resonance peaks occur at about 6 and 12 Hz, but with interindividual physiological variations [38]. The results are consistent with other studies that indicated a maximal increase of HR in the low frequencies of vertical stimulation when compared to higher frequencies [4]. It has also been demonstrated that exposure to vertical WBV (1–30 Hz) influences the heart rate more than exposure to horizontal vibration [39]. There are even authors who consider that vertical vibration (2–20 Hz) produces cardiovascular effects similar to those normally occurring during moderate exercise [40]. Another related study highlighted the slight increase of HR in association with a minor increase of oxygen uptake (VO_2_) and blood pressure in the case of exposure to WBV at 30 Hz [41].

The action of diagonal stimuli is, however, more complex and difficult to interpret. In this situation, the resonance of the internal organs by multiaxial mechanical stimulation is nonlinear [42]. In our case, the influence of vibrations on HR for the diagonal stimulation at 25 Hz is more important than for the vertical stimulation at 35 Hz (*p* < 0.05). Other studies have also confirmed that transverse angular vibration has stronger effects on the human body than vertical vibrations alone [43].

In regard to changes in SaO_2_, our results are inconclusive, due to the non-normal distribution of data. However, we can observe a slight tendency of mean decrease of SaO_2_, especially in the case of diagonal stimuli, with a maximum effect at 25 Hz (f2d protocol). Generally, studies related to changes in this blood parameter as a result of exposure to WBV are controversial. However, the occurrence of increased metabolic demand for oxygen and peripheral blood flow effects is suggested, especially for low stimulation frequencies and in the case of side-alternating WBV [44]. In addition, some authors cite a minor decrease in SaO_2_ of about 1%, with negligible clinical meaningfulness, in the case of WBV application, with a frequency of 25 Hz in patients with COPD [45].

For RR, we also could not apply the ANOVA analysis under non-normal data distribution. Only as an observation, there was a slight increase in mean RR after exposure to all protocols of WBV. The maximum mean increase was again recorded in the case of diagonal stimuli with a frequency of 25 Hz (f2d protocol).

Next, we analyzed the dynamics of the TV during the tests (Figure 2), and we highlighted its statistically significant increases after exposure to all WBV protocols. Significant statistical differences between the effects of certain stimulation protocols (Table 4) indicate a specific pattern of maximal effects. Thus, the mean increases of TV were higher under the action of diagonal stimuli, with a maximum recorded at the frequency of 15 Hz (f1d protocol). The scientific literature is brief or inconsistent in regard to this phenomenon. For example, some authors underlined, in animal studies on cats and rabbits, that TV decreases by 10–15% after exposure to longitudinal sternal vibration at a high frequency of 100 Hz [46,47].

For VC and FVC (Figure 3 and Figure 4), we put into evidence the main effect of time, WBV exposure, and interactions (time*WBV), at *p* < 0.05, with a slight average increase of both parameters for all protocols (Table 3). The maximum effects are mainly recorded after exposure to diagonal vibrations, with a maximum of mean increase at the frequency of 25 Hz (f2d protocol). The mean increase was higher for VC when comparing to FVC in all scenarios of WBV exposure, except for the f2v protocol, when the values were equal. It can be observed that the mean values of VC are slightly higher than those of FVC for each testing protocol, both before and after exposure to WBV. However, exposure to WBV lightly increases the mean differences between the two parameters, due to the more marked increase in VC versus FVC. Specifically, the mean gap is maximal (0.11 L) after f2d and f3d protocols. In clinical practice, there is little or no difference between VC and FVC in normal subjects, with VC usually being a little higher than FVC at rest [48]. The difference between the forced and slow vital capacity is related to the degree of hyperinflation and can predict its intensity [49]. Moreover, in pathological conditions (such as COPD or asthma patients), VC is greater than FVC, and the difference between the two mentioned parameters could be explained in terms of airflow limitation, small airway collapse, and gas trapping [48,50,51].

From an inferential point of view, however, the results are more difficult to interpret after the post hoc analysis, because only the results of certain protocols differ from others in statistical terms (Table 4). Practically, the f2d protocol is noticeably different from all others, except f3d for VC and f1d for FVC (*p* < 0.05). For the comparative analysis of these results, we could identify only a few studies focusing on the acute physiological effects of WBV on VC and FVC. Thus, most research of this type has been conducted on animals or targeted the chronic effects of exposure to vibration programs associated with physical exercise in various pathologies or older adults. For example, one recent research performed on older subjects that participated in resistance training on the WBV platform for three months showed an increase in respiratory muscles’ strength and incremental increases in chest-wall total volume [52]. Instead, in an earlier study focused on the effect of whole-body vertical vibration on respiration in human subjects, in the case of exposure for only 4 min at low-frequency stimuli (2–7 Hz), the subjects’ vital capacity was not changed [53].

Another analysis refers to the changes of FEV_1_ after the exposure to WBV (Figure 5). The observed changes are not very extensive, but they appear during all protocols (*p* < 0.05) and converge with the evolution of VC and FVC. Again, the exposure to WBV with diagonal stimuli exerts the higher influence on FEV_1_, with maximal effects for diagonal stimulation at 25 Hz (f2d protocol). However, the post hoc analysis reveals statistical differences only between the results of the f1v and f2d protocols, which limit the extrapolation of the results (Table 4). The interpretation of the results in the context of other research remains difficult, because, as we have already mentioned, most similar studies refer to the effects of chronic exposure to WBV in patients with pulmonary pathology. For example, limited evidence suggested that WBV training might enhance pulmonary function in COPD patients regarding the change of FEV_1_ (% predicted) [54].

The last parameter investigated was MVV (Figure 6). In relation to this parameter, the results indicated the main effect of time, WBV exposure, and interactions (time*WBV), at *p* < 0.05, but no effects were found between the six protocols (Table 3 and Table 4). However, the results suggest the same pattern of change of MVV in the group of subjects under WBV programs, with a higher mean increase in the case of exposure to diagonal stimuli, and with a maximum of mean increase at 25 Hz (f2d protocol). For MVV, a similarity of dynamics with VC, FVC, and FEV_1_ is observed, which can be explained by the existence in physiological conditions of a high correlation between MVV and FEV_1_ [55].

On the basis of the results provided by our research, it is noted that, overall, the growth pattern of the spirometry indicators is common for TV, VC, FVC, FEV_1_, and MVV, with maximum effects recorded on diagonal stimulation. In the case of the diagonal stimulation for TV, the maximum effects are observed at 15 Hz (f1d protocol), while for VC, FVC, FEV_1_, and MVV at 25 Hz (f2d protocol). Since TV is a physical component included in the estimation of VC, FVC, FEV_1_, and MVV, it becomes obvious that an important part of the increase in these parameters is caused by the increase in TV. Our findings can be clearly interpreted from several perspectives. Thus, from a biophysical point of view, the vibrations of the pulmonary structures cause mechanical stress at the level of a biphasic medium of the lung with two media: low-density compressible gas (air from the respiratory tract) and high-density incompressible soft tissues, with viscoplastic properties [56]. Obviously, the therapeutic forced vibrations penetrate the entire thorax, causing alveolar and pleural pressure oscillations [56,57]. As a consequence, the behavior of the pulmonary tissue under the action of vibrations and the residual effects can be analyzed from the perspective of the elastoplastic rheological models with viscous properties [58].

Animal experimental studies have highlighted that high-frequency vibrations applied on the thorax may influence the TV, due to the pendular flow oscillation between lung regions [59]. Moreover, vibration augments the expiratory flow and may help mobilize mucus [19,60]. Clearly, the entire body’s vibrations, which are also transmitted to the pulmonary structures, cause local effects, with implications for the ventilator parameters. This has been demonstrated by other authors with regard to the occurrence of airway pressure oscillations at the same frequency as the mechanical vibrations, especially when using high vibration amplitudes. The explanations provided in this case are related to the excitation of the intrapulmonary receptors [57]. It is important to mention that the results of some animal studies on dogs assert that high-frequency vibrations exerted on the chest wall do not significantly influence the functioning of the diaphragm muscle, phrenic motoneurons, and medullary inspiratory neurons [61]. Likewise, other animal studies suggest that high-frequency vibrations (40 Hz) do not significantly modify the stretch reflexes in external intercostal muscles and, as a result, there are no significant changes of the mechanical behavior of the respiratory system [62].

It has been proven that there is a certain dynamic of lung vibration during ventilation [63]. Thus, it seems highly plausible that external vibrations significantly interact with the pulmonary tissue and modify the ventilator pattern. Furthermore, it seems interesting to consider whether this situation might be due to the presence of acute remnant effects of vibrations on the pulmonary alveoli (possible at the surfactant level by modifying the surface tension distribution in alveoli) and on other pulmonary parenchymal structures. A study conducted on newborns revealed the positive effects of vibrations with frequencies of 10–70 Hz on improving surfactant function of diffusion and adsorption of surfactant molecules to the air–liquid interface in the alveoli [64]. In addition, at least in the case of mechanically ventilated patients, the lung vibrations manually executed an increase in the alveolar recruitment and ventilation/perfusion matching [65]. Last but not least, the systemic effects of WBV on the human body can be interpreted from a neuroendocrine perspective. Thus, several studies have shown an increase in plasma levels of IL-10, IGF-1 glucocorticoids, and catecholamines after WBV exposure [66,67].

The maximal ventilatory changes can also be correlated with the resonance frequencies under the action of vibratory stimuli of the tissues involved in the breathing process that cause neuromuscular reflex responses and improve neuromuscular performance [68]. Returning to the interpretation of the effects of the diagonal vibrations on the human body, we can appeal to the biaxial model of the decomposition of diagonal forces in relation to two perpendicular axes. Therefore, we can understand that human responses to multi-axis vibration are highly nonlinear and cross-coupled, with resonance frequencies decreasing with increasing magnitude of vibration [42]. Another experimentally demonstrated fact is that angular vibrations determine the perception of some increased psychophysical effects, as a function of both the frequency and the intensity of the stimulus vibrations [69]. Moreover, it seems that the transverse angular vibration exerts a higher influence on human neuromotor reactions than the vertical vibrations [43].

In this research, we put into evidence the maximal effects of the diagonal vibrations on the cardiorespiratory parameters, especially at the frequency of 25 Hz. It is considered that the diagonal mode of vibrations, with a maximal shear effect on the connective tissue structures, corresponds to the natural motion patterns (walking and running) [31]. In this way, we can explain the maximal recorded effects at the level of cardiorespiratory indicators in the case of the diagonal stimulation. In present, there is little evidence as to the optimal vibration training protocol for different target groups in terms of minimal risks and maximum benefits for the patients [70]. This study also reveals the potential for future research about the clinical efficiency of mixed WBV programs that alternate the exposure of subjects to different frequencies of diagonal and vertical stimuli, possibly with the association of physical exercise programs. Practically, such programs may become useful in the context of prophylactic and rehabilitation treatment of patients with restrictive or obstructive pulmonary disorders to the extent that they are not detrimental and determine maximum cumulative cardiorespiratory effects.

To our knowledge, this is the first human study to compare the acute effects of WBV that was applied according to different protocols, including in respect to the frequency and direction of the stimuli, on cardiorespiratory parameters. In addition, as a novelty element, a comparative analysis of the WBV effects with vertical versus diagonal stimuli was performed. However, there are some bibliographic references related to studies on the effects of manual vibrations on the respiratory system, but using low-frequency stimulation [19,20].

A limitation of the study derives from the size of the sample and the absence of a control group. We did not perform a comparative analysis of the results by sex, given the relatively small number of subjects and the large number of variables considered. In such situations, the application of statistical methods to subgroups of subjects increases the risk of type I error [71].

Moreover, we did not consider it appropriate to test different amplitudes of vibrations, because there would have been too much of an increase in the volume of recorded data, thus negatively affecting the robustness of the statistical analysis. From a multitude of data obtained, we extracted only those that generated inferential conclusions. Of course, there is some degree of uncertainty regarding the statistically insignificant results, and it is not recommended to consider the lack of generalization of some results as indicating no effects on investigated parameters. Rather, this indicates a lack of evidence against the null hypothesis between the groups of subjects.

The present study did not set as its objective the determination of the harmful effects of WBV on human health, but only the acute physiological effects on the cardiorespiratory parameters. Moreover, such an intention is difficult to achieve, given the risks to participants and the difficulties of interpreting such results. On the other hand, starting from the amplitude of the observed effects, presumptions can be made about the usefulness and greater efficiency of certain WBV stimulation protocols within the therapeutic procedures. In this sense, the continuation of research for various pathologies and taking into account other categorical variables become very attractive.

The strength of this research lies in the way in which the six WBV exposure protocols of the subjects were selected and applied, alternately using stimuli with different frequencies and directions. Moreover, the comparative analysis of the effects of vertical and diagonal stimuli, at three usual frequencies, offers a new perspective on the possibility of using preferential variants to obtain maximum physiological effects to maintain and improve the health of individuals.

## 5. Conclusions

In the context of rejecting the null hypotheses at the 5% significance level, the results of our study revealed significantly higher values for HR, TV, VC, FVC, FEV_1_, and MVV after the WBV exposure, as compared with the initial assessments (before the WBV exposure). The mentioned cardiorespiratory parameters are significantly influenced by both the frequency and direction of the stimuli, and certain protocols of WBV are noticeable for their distinct effects. Thus, WBV with diagonal stimuli causes wider effects than WBV with vertical ones in most situations. When applying WBV with diagonal stimuli, maximum effects are observed at the 25 Hz frequency for HR, VC. FVC, and FEV_1_, while, for TV, the maximum effects are reported at 15 Hz. Therefore, in order to achieve maximum acute cardiorespiratory effects by exposure to WBV, protocols based on specific combinations of stimuli, such as frequency and direction of stimulation, may be proposed. In this way, depending on the clinical context, professionals may be able to optimize the use of vibration platforms as evidence-based interventions.

## Figures and Tables

**Table 1:** Statistic indicators for anthropometric parameters in the experimental group (*n* = 26).

Variable	Age (Years)	H (cm)	W (kg)	BMI (kg/m^2^)
Mean	20.73	167.06	60.69	21.70
SD	1.15	9.90	9.18	1.72
CV(%)	5.55	5.93	15.13	7.93

W, weight; H, height; SD, standard deviation; CV, coefficient of variation.

**Table 2:** Statistic indicators for investigated parameters in the experimental group (*n* = 26).

Variable	HR (Beats/min)		SaO_2_ (%)		RR (Breaths/min)		TV (L)		VC (L)		FVC (L)		FEV_1_ (L)		MVV (L/min)	
f1 (15 Hz), vertical stimuli																
	B	A	B	A	B	A	B	A	B	A	B	A	B	A	B	A
Mean	69.50	76.38	96.12	95.65	15.46	17.35	0.44	0.47	4.04	4.15	3.95	4.04	3.33	3.40	140.52	143.22
SD	7.33	7.58	0.77	0.75	0.99	1.09	0.06	0.07	0.57	0.58	0.57	0.57	0.51	0.51	18.99	19.27
CV (%)	10.55	9.92	0.80	0.78	6.40	6.28	13.64	14.89	14.11	13.98	14.43	14.11	15.32	15.00	13.51	13.45
f2 (25 Hz), vertical stimuli																
	B	A	B	A	B	A	B	A	B	A	B	A	B	A	B	A
Mean	69.92	75.23	95.77	95.35	15.69	17.08	0.44	0.45	4.04	4.09	3.96	4.01	3.35	3.39	141.32	142.98
SD	7.35	7.19	0.71	0.85	1.01	1.06	0.06	0.06	0.56	0.57	0.55	0.55	0.50	0.50	18.69	18.74
CV (%)	10.51	9.56	0.74	0.89	6.44	6.21	13.64	13.33	13.86	13.94	13.89	13.72	14.93	14.75	13.23	13.11
f3 (35 Hz), vertical stimuli																
	B	A	B	A	B	A	B	A	B	A	B	A	B	A	B	A
Mean	70.19	74.42	95.92	95.38	15.58	16.85	0.44	0.45	4.03	4.11	3.96	4.03	3.33	3.39	140.74	142.84
SD	6.47	6.76	0.84	1.10	1.06	0.92	0.07	0.06	0.56	0.57	0.54	0.55	0.48	0.49	18.08	18.43
CV (%)	9.22	9.08	0.88	1.15	6.80	5.46	15.91	13.33	13.90	13.87	13.64	13.65	14.41	14.45	12.85	12.90
f1 (15 Hz), diagonal stimuli																
	B	A	B	A	B	A	B	A	B	A	B	A	B	A	B	A
Mean	70.19	76.04	96.04	95.27	15.58	17.35	0.44	0.53	4.04	4.17	3.96	4.08	3.34	3.44	140.86	144.5
SD	6.38	6.30	0.87	1.04	1.17	1.02	0.06	0.06	0.56	0.58	0.56	0.57	0.51	0.51	18.95	19.16
CV (%)	9.09	8.29	0.91	1.09	7.51	5.88	13.64	11.32	13.86	13.91	14.14	13.97	15.27	14.83	13.45	13.26
f2 (25 Hz), diagonal stimuli																
	B	A	B	A	B	A	B	A	B	A	B	A	B	A	B	A
Mean	70.58	78.23	96.15	95.23	15.50	18.08	0.44	0.48	4.04	4.22	3.96	4.11	3.33	3.45	140.6	145.30
SD	6.59	6.43	0.78	0.86	0.99	0.98	0.06	0.06	0.55	0.59	0.53	0.54	0.47	0.48	17.71	17.89
CV (%)	9.34	8.22	0.81	0.90	6.39	5.42	13.64	12.50	13.61	13.98	13.38	13.14	14.11	13.91	12.60	12.31
f3 (35 Hz), diagonal stimuli																
	B	A	B	A	B	A	B	A	B	A	B	A	B	A	B	A
Mean	70.50	76.69	96.42	95.77	15.81	17.62	0.44	0.46	4.03	4.16	3.94	4.05	3.32	3.41	140.34	143.55
SD	6.40	5.89	0.64	0.65	1.20	0.94	0.06	0.06	0.57	0.67	0.56	0.57	0.49	0.50	18.39	18.8
CV (%)	9.08	7.68	0.66	0.68	7.59	5.33	13.64	13.04	14.14	16.11	14.21	14.07	14.76	14.66	13.10	13.10

HR, heart rate; SaO_2_, arterial oxygen saturation; RR, respiratory rate; TV, tidal volume; VC, vital capacity; FVC, forced vital capacity; FEV_1_, forced expiratory volume at 1 s; MVV, maximum voluntary ventilation; B, before; A, after; f, frequency of stimuli; SD, standard deviation; CV, coefficient of variation.

**Table 3:** Results of Tests of Within-Subjects Effects for all subjects, comparing the conditions “before and after” different types of WBV exposure.

Parameter	Effect	Type III Sum of Squares	df	Mean Square	F	*p*-Value	Partial Eta Squared	Observed Power
HR	WBV	148.362	5	29.672	2.754	0.021	0.099	0.812
	Time	2826.029	1	2826.029	2074.638	0.000	0.988	1
	Time*WBV	93.413	3.093	30.2	18.244	0.000	0.422	1
TV	WBV	0.063	3.537	0.018	72.030	0.000	0.742	1
	Time	0.094	1	0.094	740.631	0.000	0.967	1
	Time*WBV	0.048	2.808	0.017	112.299	0.000	0.818	1
VC	WBV	0.009	2.291	0.004	9.539	0.000	0.276	0.985
	Time	0.058	1	0.058	483.477	0.000	0.951	1
	Time*WBV	0.008	1.193	0.007	11.611	0.001	0.317	0.939
FVC	WBV	0.006	5	0.001	6.602	0.000	0.209	0.997
	Time	0.047	1	0.047	1455.686	0.000	0.983	1
	Time*WBV	0.005	5	0.001	47.588	0.000	0.656	1
FEV_1_	WBV	0.005	5	0.001	2.648	0.026	0.096	0.794
	Time	0.047	1	0.047	1164.269	0.000	0.979	1
	Time*WBV	0.005	5	0.001	39.556	0.000	0.613	1
MVV	WBV	0.003	5	0.001	1.961	0.089	0.073	0.645
	Time	0.033	1	0.033	1838.704	0.000	0.987	1
	Time*WBV	0.004	5	0.001	37.697	0.000	0.601	1

Note: df, the degrees of freedom in the source.

**Table 4:** Results of Bonferroni post hoc comparisons.

Parameter	Time	WBV					
		f1v	f2v	f3v	f1d	f2d	f3d
HR	B and A (*p* < 0.01)	-	-	f2d (*p* < 0.02)	-	f3v (*p* < 0.02)	-
TV	B and A (*p* < 0.01)	f3v (*p* < 0.01); f1d (*p* < 0.01)	f1d (*p* < 0.01); f2d (*p* < 0.01)	f1v (*p* < 0.01); f1d (*p* < 0.01); f2d (*p* < 0.01); f3d (*p* < 0.04);	f1v (*p* < 0.01); f2v (*p* < 0.01); f3v (*p* < 0.01); f2d (*p* < 0.01); f3d (*p* < 0.01)	f2v (*p* < 0.01); f3v (*p* < 0.01); f1d (*p* < 0.01); f3d (*p* < 0.01)	f3v (*p* < 0.04); f1d (*p* < 0.01); f2d (*p* < 0.01)
VC *	B and A (*p* < 0.01)	f2d (*p* < 0.01)	f1d (*p* < 0.01); f2d (*p* < 0.01)	f1d (*p* < 0.01); f2d (*p* < 0.01)	f2v (*p* < 0.01); f3v (*p* < 0.01); f2d (*p* < 0.01)	f1v (*p* < 0.01); f2v (*p* < 0.01); f3v (*p* < 0.01); f1d (*p* < 0.01)	-
FVC *	B and A (*p* < 0.01)	f2d (*p* < 0.01)	f2d (*p* < 0.01)	f1d (*p* < 0.04); f2d (*p* < 0.01)	f3v (*p* < 0.04)	f1v (*p* < 0.01); f2v (*p* < 0.01); f3v (*p* < 0.01); f3d (*p* < 0.01);	f2d (*p* < 0.01)
FEV_1_ *	B and A (*p* < 0.01)	f2d (*p* < 0.05)	-	-	-	f1v (*p* < 0.05)	-
MVV *	B and A (*p* < 0.01)	-	-	-	-	-	-

Note: * calculated based on natural log-transformed data; B, before; A, after; *p*, level of statistical significance.

## Data Availability

The data are available upon request from the corresponding author. All data relevant to the study are included in the article.
